# Comparison of Microperimetry and Static Perimetry for Evaluating Macular Function and Progression in Retinitis Pigmentosa

**DOI:** 10.1016/j.xops.2024.100582

**Published:** 2024-07-20

**Authors:** Masatoshi Fukushima, Yan Tao, Sakurako Shimokawa, Huanyu Zhao, Shotaro Shimokawa, Jun Funatsu, Takahiro Hisai, Ayako Okita, Kohta Fujiwara, Toshio Hisatomi, Atsunobu Takeda, Yasuhiro Ikeda, Koh-Hei Sonoda, Yusuke Murakami

**Affiliations:** 1 Department of Ophthalmology, Graduate School of Medical Sciences, Kyushu University, Fukuoka, Japan; 2 Department of Ophthalmology, Fukuoka University Chikushi Hospital, Fukuoka, Japan; 3 Department of Ophthalmology, Graduate School of Medicine, Oita University, Oita, Japan; 4 Department of Ophthalmology, Faculty of Medicine, University of Miyazaki, Miyazaki, Japan

**Keywords:** Disease progression, Microperimetry, OCT, Retinitis pigmentosa, Static perimetry

## Abstract

**Purpose:**

To compare the usefulness of microperimetry and static automated perimetry in patients with retinitis pigmentosa (RP), using macular anatomical metrics as a reference.

**Design:**

Prospective observational study.

**Participants:**

Forty-eight eyes of 48 patients with RP in Kyushu University Hospital who underwent microperimetry-3 (MP-3) and Humphrey Field Analyzer (HFA) 10-2 testing ≥3 times during ≥2 years were included.

**Methods:**

Macular anatomy (ellipsoid zone [EZ] length) was assessed by OCT, and macular function was assessed by MP-3 (mean retinal sensitivity at radii 2°, 4°, and 8°) and HFA10-2 program (mean retinal sensitivity at radii 2°, 4°, and 8°). Correlations between functional and anatomical parameters were analyzed cross sectionally at baseline and longitudinally by comparing the rate of progression.

**Main Outcome Measures:**

Correlation coefficients between anatomical and functional metrics.

**Results:**

The mean age at baseline was 50.1 ± 12.3 years, and the mean follow-up period was 2.8 ± 0.7 years. At baseline, EZ length was significantly correlated with MP-3 mean retinal sensitivity at radii 2°, 4°, and 8° (Spearman’s ρ = 0.65, 0.84, 0.89; all *P* < 0.005) and HFA10-2 mean retinal sensitivity at radii 2°, 4°, and 8° (Spearman’s ρ = 0.61, 0.73, 0.78; all *P* < 0.005). Longitudinal analysis showed that the slope of EZ length (−88.92 μm/year) was significantly correlated with the slope of MP-3 retinal sensitivity at 8° radius (−0.62 decibels [dB]/year; Spearman’s ρ = 0.31, *P*=0.03) and the slope of HFA retinal sensitivity at 8° radius (−0.60 dB/year; Spearman’s ρ = 0.43, *P* < 0.005).

**Conclusions:**

Both MP-3 and HFA values were cross sectionally well-correlated with EZ length in patients with patients; however, these associations became weaker in the longitudinal analysis. This highlights the need for researchers to explore additional or more sensitive parameters to better monitor RP progression.

**Financial Disclosure(s):**

Proprietary or commercial disclosure may be found in the Footnotes and Disclosures at the end of this article.

Retinitis pigmentosa (RP) is a group of inherited retinal degeneration disorders characterized by the symptoms of night blindness, visual field constriction, and a subsequent decline of visual acuity in the later stage.^1^ Because a loss of central vision substantially attenuates the quality of life among individuals with RP, a precise evaluation of macular function is important to explain the disease status and progression to patients as well as to evaluate the efficacy of potential therapeutics in clinical trials.^2^, ^3^, ^4^, ^5^ Therefore, there is a significant need for sensitive measures that can reliably detect the disease progression and therapeutic effects in patients with RP.

Macular function and progression of the central vision loss in RP are evaluated by microperimetry (MP),^6^, ^7^, ^8^, ^9^, ^10^, ^11^, ^12^, ^13^ static automated perimetry (SAP),^14^, ^15^, ^16^, ^17^, ^18^, ^19^, ^20^, ^21^, ^22^, ^23^, ^24^, ^25^ OCT,^13^^,^^23^, ^24^, ^25^, ^26^, ^27^ fundus autofluorescence,^28^, ^29^, ^30^, ^31^ and electroretinography.^32^ In particular, retinal sensitivity values provided by MP or SAP are frequently used as functional outcome measures in clinical studies and trials of patients with RP; however, it has not been fully elucidated which measurement method is better for use in clinical trials or daily practice, and there is a need for comparative studies to be conducted.

There are some advantages and disadvantages to both MP and SAP. Microperimetry directly projects light stimuli onto the retina while simultaneously imaging the fundus with auto-tracking, which facilitates the precise measurement of retinal sensitivity at specific retinal points.^6^^,^^7^ On the other hand, MP is reported to require a longer test time than SAP such as a Humphrey Field Analyzer (HFA).^7^ Humphrey Field Analyzer has the advantage of being used as the primary outcome in clinical trials of glaucoma^33^, ^34^, ^35^ and short measurement time,^7^ and HFA devices are widely distributed in ophthalmology facilities in Japan. However, SAP has the disadvantage of lower accuracy in evaluating macular function with unstable fixation patients.^36^

In patients with RP, MP including Macular Integrity Assessment (MAIA), microperimetry-1, and microperimetry-3 (MP-3) is frequently used to assess macular function in clinical studies and trials, because MP with real-time fundus tracking is advantageous for those with fixation issues.^8^, ^9^, ^10^, ^11^, ^12^ Static automated perimetry such as Octopus and HFA is also used in clinical trials for RP but less frequently.^2^^,^^14^ It has been shown that retinal sensitivity values measured by both MP (MAIA, microperimetry-1, and MP-3) and SAP (Octopus and HFA) are well correlated with retinal anatomical parameters.^7^^,^^8^^,^^11^^,^^13^^,^^15^^,^^16^^,^^23^, ^24^, ^25^, ^26^ Two previous studies directly compared the performance of MP-3 and HFA in patients with RP, and MP-3 showed a better structure–function relationship^7^ and better test–retest reproducibility^6^ than HFA. However, there has been no report directly comparing the usefulness of MP and SAP for evaluating RP progression. Therefore, in the present study we compared the performance of MP-3 and HFA10-2 in assessing RP macular status and progression using macular anatomical metrics as a reference.

## Methods

### Ethics Statement

This study was approved by the institutional review board of Kyushu University Hospital (Fukuoka, Japan) and was conducted in accordance with the tenets of the Declaration of Helsinki on Biomedical Research Involving Human Subjects (ethics approval number: 22357). Written informed consent was obtained from all subjects after a thorough explanation of the nature of the study and its possible consequences.

### Study Design and Patients

This prospective, single-center, observational study was conducted to evaluate the macular function and progression in patients with typical RP. The diagnosis of typical RP was based on a history of night blindness; visual field constriction or ring scotoma; and markedly reduced or nonrecordable a- and b-wave amplitudes in electroretinography testing, in addition to characteristic ophthalmoscopic findings (e.g., bone spicule-like pigment clumping in the midperipheral and peripheral retina and attenuation of retinal vessels).

Inclusion criteria of this study were (1) diagnosis of typical RP; (2) age of >20 years; (3) logarithmic minimum angle of resolution best-corrected visual acuity (BCVA) better than 1.0; and (4) ellipsoid zone (EZ) length of ≥500 μm. Participants underwent BCVA measurement and assessment by MP-3 (NIDEK), HFA (Carl Zeiss Meditec), and OCT (NIDEK) on the same day at some time point between October 2016 and March 2019, and then were followed-up with annual examinations that included all these measures. The examination results of the right eye of each patient were used for the analyses.

Sixty-seven eyes of 67 patients were included, and 48 eyes of 48 patients with RP who underwent MP-3 and HFA10-2 testing ≥3 times during ≥2 years were analyzed.

### Visual Acuity

The BCVA was measured in decimal units with full subjective refraction using a Landolt ring chart at 5 m for all patients at each visit. The decimal acuities were converted into a logarithmic minimum angle of resolution for statistical evaluation.

### Visual Field Testing

**Figure 1:**
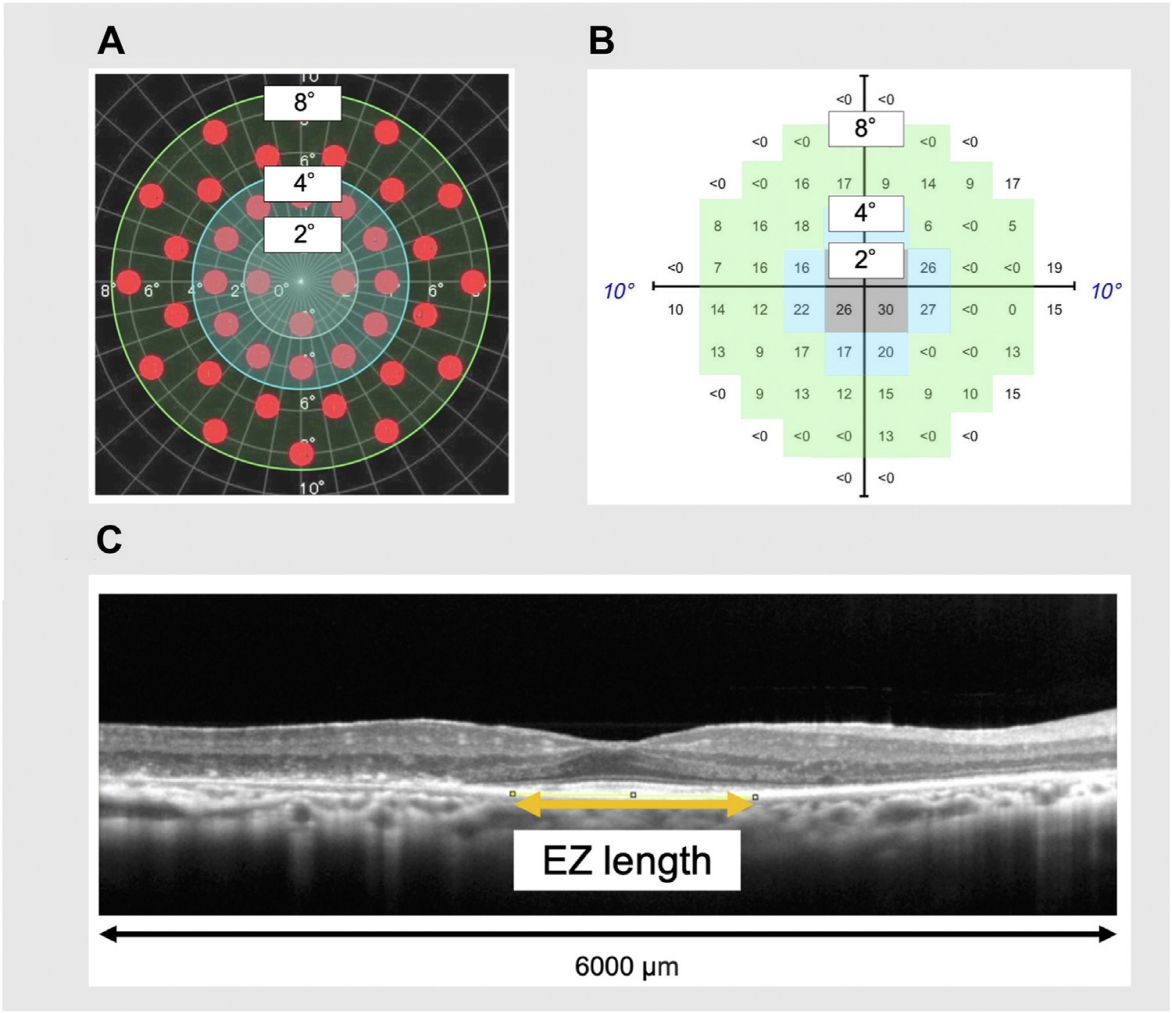
Methods used to evaluate the macular function with MP-3 and HFA and the macular structure with OCT. **A,** In MP-3 data analysis, retinal sensitivity loci was divided into the area of 2° radius (central 4 points), 4° radius (central 16 points), and 8° radius (central 40 points). **B,** In HFA data analysis, retinal sensitivity loci was divided into the area of 2° radius (central 4 points), 4° radius (central 12 points), and 8° radius (central 52 points). **C,** The EZ length in OCT image was measured as indicated by the orange arrow. EZ = ellipsoid zone; HFA = Humphrey Field Analyzer; MP-3 = microperimetry-3.

The MP-3 examination was performed to measure the retinal sensitivity using a unique measurement program with 40 points symmetrically placed within an 8° radius from the center, as shown in Figure 1A. The stimulus dynamic range for the MP-3 was 0-34 decibels (dB). The background luminance was 31.4 asb, and the maximum luminance was 10 000 asb. The MP-3 examination was carried out using the standard Goldmann III stimulus size with a duration of 200 ms and the thresholding algorithm was on the basis of a 4–2 full threshold strategy. The mean retinal sensitivities of the central 4, 16, and 40 points were used as the mean sensitivity at the radii of 2°, 4°, and 8° (Fig 1A).

All patients underwent automated static perimetry with the central 10-2 Swedish Interactive Threshold Algorithms—Fast program with 64 measurement points within 10° from the center. The stimulus dynamic range for the HFA was 0 to 50 dB. The background luminance was 31.5 asb, and the maximum luminance was 10 000 asb. The HFA examination was carried out using the standard Goldmann III stimulus size with a duration of 200 ms. The thresholding algorithm was Double Bracketing Strategy, which strategy was based on a 4–2 full threshold strategy, with maximum likelihood estimation. The mean retinal sensitivities in the central 4, 12, and 52 points were used as the mean sensitivity at the radii of 2°, 4°, and 8° (Fig 1B).

In our study, 44 of 48 patients (91.7%) had received ≥2 HFA tests before entering this study. Three patients had undergone 1 HFA test, and only 1 patient had never had a HFA test. Therefore, the effect of learning effect is considered to be relatively small.

### OCT

The RS-3000 OCT system was used for retinal imaging, and the horizontal OCT scans through the fovea with a radius of 3000 μm from the center were analyzed. These horizontal scans were used to evaluate the width of the residual EZ line as the anatomical evaluation metric. The EZ line was defined as the line between the points of EZ disappearance in the horizontal 6000 μm OCT scans through the fovea. The EZ length was defined as the length of the straight line drawn between the points of EZ disappearance (Fig 1C). Using ImageJ software on an external personal computer to measure the pixel count, the EZ length was calculated based on the image width of 6000 μm; M.F. and Y.T. were trained by Y.M. on this segmentation prior to measurement. After the measurement by Y.T., the EZ measurement was verified by M.F.

### Statistical Analysis

The R Studio software package, version 2022.02.1+461 "Prairie Trillium" (RStudio PBC), was used to perform all the statistical analyses. In order to calculate the regression coefficients for each patient, we used simple linear regression analyses with time as the independence variable and change of EZ length or retinal sensitivity as dependence variable. Correlations coefficients between the slope of EZ length and retinal sensitivities were calculated in 48 patients with RP. The relationships between retinal sensitivity and structural parameters were analyzed using the Spearman's rank correlation coefficient. *P* values <0.05 were considered significant.

## Results

### Patient Baseline Characteristics

The demographic data of the study subjects are summarized in Table 1. The mean age was 50.1 ± 12.3 years, the mean BCVA was 0.04 ± 0.19 logarithmic minimum angle of resolution, the mean follow-up period was 2.8 ± 0.7 years, and the mean test interval period was 1.2 ± 0.4 years. Thirty-three (68.6%) of the eyes were examined 3 times and 15 (31.3%) of the eyes were examined 4 times during the follow-up period. The modes of inheritance as obtained from clinical records were as follows: autosomal dominant in 4 (8.3%) patients, autosomal recessive in 12 (25%), X-linked recessive in 1 (2.1%), and sporadic in 31 (64.6%). The causative genes were determined in the previous genomic sequence analysis of 83 RP causative genes^37^ as follows: eye shut homolog in 10 patients (20.1%), usherin in 3 patients (6.3%), pre-mRNA processing factor 8 in 2 patients (4.2%), retinitis pigmentosa 1 in 1 patient (2.1%), rhodopsin in 1 patient (2.1%), retinitis pigmentosa 1-like 1 in 1 patient (2.1%), and retinitis pigmentosa GTPase regulator in 1 patient (2.1%), and the causative genes were not determined in 29 patients (60.4%).

**Table 1:** Characteristics of the 48 Eyes of the 48 Patients

Parameters	All Patients (n = 48)
Age, mean (±[std]), yrs	50.1 ± 12.3
Age at onset, mean (±[std]), yrs	28.5 ± 15.7
Male, n (%)	24 (50)
BCVA, mean (±[std]), logMAR	0.04 ± 0.19
BCEA 68%, mean (±[std]), deg^2^	1.06 ± 0.64
BCEA 95%, mean (±[std]), deg^2^	2.84 ± 1.72
Follow-up period, mean (±[std]), yrs	2.8 ± 0.7
Test interval, mean (±[std]), yrs	1.2 ± 0.4
Number of examinations	
3 times, n (%)	33 (68.6)
4 times, n (%)	15 (31.3)
Inheritance mode, n (%)∗	
Autosomal dominant	4 (8.3)
Autosomal recessive	12 (25)
X-linked recessive	1 (2.1)
Sporadic	31 (64.6)
RP causative gene, n (%)†	
*EYS*	10 (20.1)
*USH2A*	3 (6.3)
*PRPF8*	2 (4.2)
*RP1*	1 (2.1)
*RHO*	1 (2.1)
*RP1L1*	1 (2.1)
*RPGR*	1 (2.1)
Not determined	29 (60.4)

BCEA = bivariate contour ellipse area; BCVA = best-corrected visual acuity; *EYS* = eye shut homolog; logMAR = logarithmic minimum angle of resolution; *PRPF8* = pre-mRNA processing factor 8; *RHO* = rhodopsin; RP = retinitis pigmentosa; *RP1* = retinitis pigmentosa 1; *RP1L1* = retinitis pigmentosa 1-like 1; *RPGR* = retinitis pigmentosa GTPase regulator; *std* = standard deviation; *USH2A* = usherin.

∗ The inheritance mode was determined clinically by family history obtained from the patients’ medical charts.

† These genes were determined in the previous genomic sequence analysis of 83 RP causative genes (Koyanagi, et al 2019). Inheritance pattern was presumed based on the result of sequencing.

### Cross-Sectional Baseline Correlation between Functional and Anatomical Metrics in Patients with RP

We next analyzed the cross-sectional relationships between MP-3 and HFA values and EZ lengths using the baseline visit data. The results are summarized in Table 2. Mean retinal sensitivities with MP-3 at the radii of 2°, 4°, and 8° were 23.3 ± 6.4 dB, 19.6 ± 7.4 dB, and 14.9 ± 7.5 dB, respectively. Mean retinal sensitivities with HFA at the radii of 2°, 4°, and 8° were 30.3 ± 6.6 dB, 28.5 ± 6.9 dB, and 21.9 ± 7.8 dB, respectively. Mean retinal sensitivities with MP-3 were lower than those with HFA. The mean value of EZ length was 3133 ± 1640 μm.

**Table 2:** The Anatomical and Functional Metrics at Baseline in Patients with Retinitis Pigmentosa

Each Metric	Mean Value ± Standard Deviation
MP-3	
Retinal sensitivity	
Radius of 2°, dB	23.3 ± 6.4
Radius of 4°, dB	19.6 ± 7.4
Radius of 8°, dB	14.9 ± 7.5
HFA	
Retinal sensitivity	
Radius of 2°, dB	30.3 ± 6.6
Radius of 4°, dB	28.5 ± 6.9
Radius of 8°, dB	21.9 ± 7.8
OCT	
EZ length, μm	3133 ± 1640

dB = decibels; EZ = ellipsoid zone; HFA = Humphrey Field Analyzer; MP-3 = microperimetry-3.

**Figure 2:**
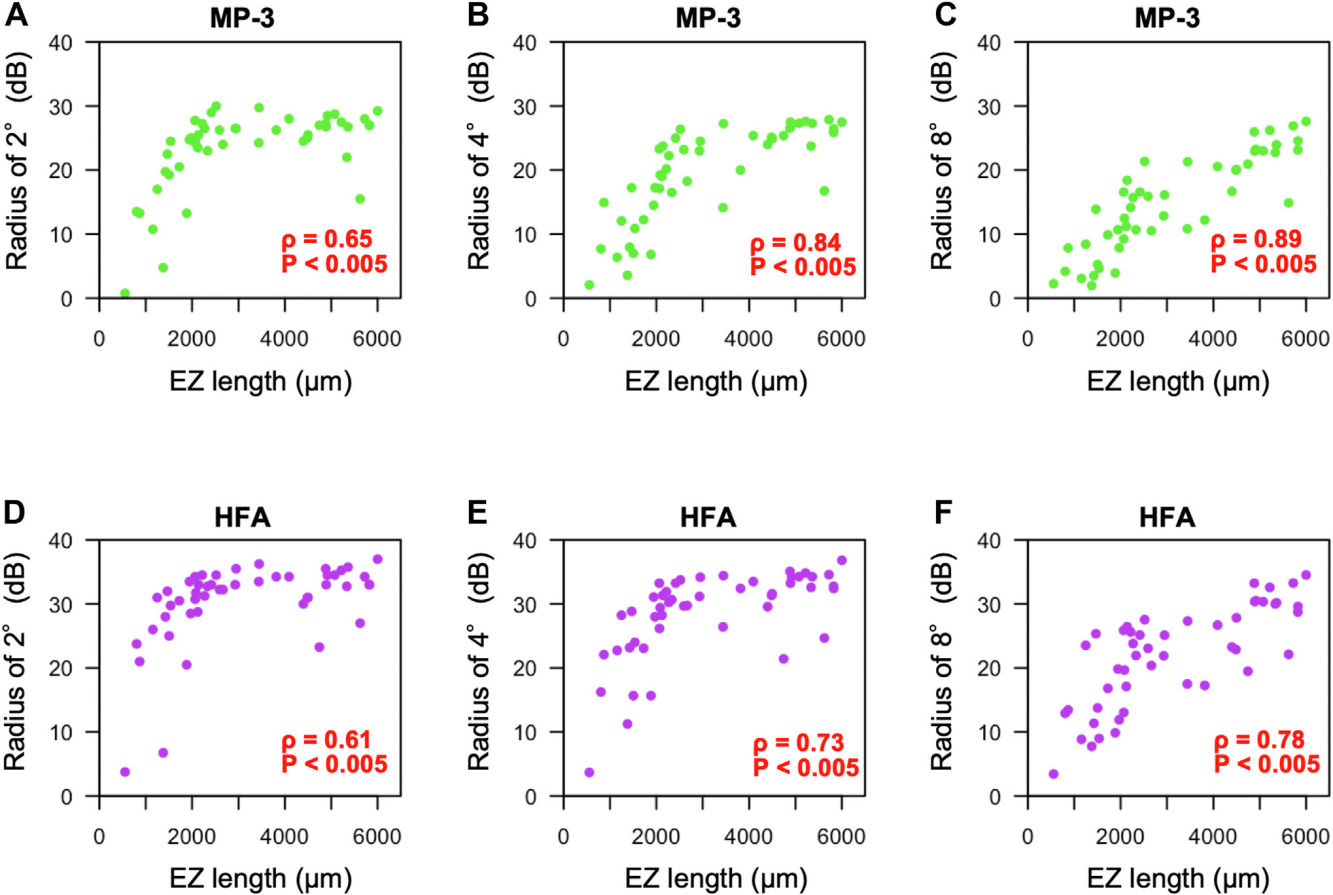
Relationships between EZ length and retinal functional parameters in cross-sectional baseline RP patient data. Scatter plots show the associations of EZ length with the mean retinal sensitivity of MP-3 at the radius 2° (**A**), 4° (**B**), and 8° (**C**), and the mean retinal sensitivity of HFA at the radius 2° (**D**), 4° (**E**), and 8° (**F**). Green dots indicate MP-3 data, and purple dots indicate HFA data. dB = decibels; EZ = ellipsoid zone; HFA = Humphrey Field Analyzer; MP-3 = microperimetry-3; RP = retinitis pigmentosa.

**Figure 3:**
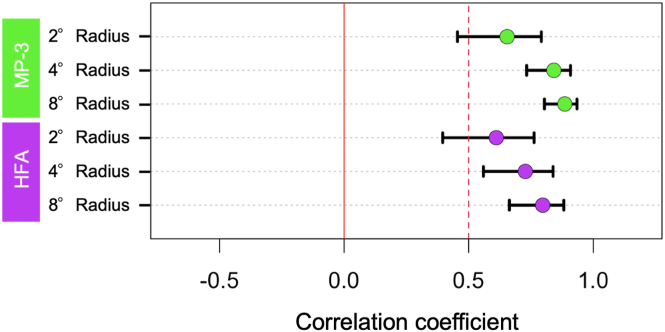
Correlation coefficients at the radii of 2°, 4°, and 8° in cross-sectional baseline data. Green and purple dots show correlation coefficients between EZ length and retinal sensitivities measured by MP-3 or HFA at the radii of 2°, 4°, and 8°. Bars show 95% confidence interval bounds for each correlation coefficient. All retinal sensitivities with either MP-3 or HFA were significantly correlated with EZ length. The correlation coefficients were analyzed by Spearman’s rank correlation test. EZ = ellipsoid zone; HFA = Humphrey Field Analyzer; MP-3 = microperimetry-3.

The correlation coefficients between EZ length and the mean retinal sensitivity at the radii of 2°, 4°, and 8° measured with MP-3 were 0.65 (*P* < 0.005), 0.84 (*P* < 0.005), 0.89 (*P* < 0.005), respectively, whereas the correlation coefficients between EZ length and the mean retinal sensitivity at the radii of 2°, 4°, and 8° measured with HFA were 0.61 (*P* < 0.005), 0.73 (*P* < 0.005), and 0.78 (*P* < 0.005), respectively (Fig 2). The correlation efficient at the corresponding area was similar for MP-3 and HFA (Fig 3).

Considering the disparity in the number of loci between the 2°, 4°, and 8° rings, we conducted an additional analysis to divide the retinal sensitivity loci into 3 areas, i.e., foveal area, parafoveal area, and perimacular area, as shown in Fig S4 (available at www.ophthalmologyscience.org). With this segmentation, cross-sectional baseline data showed that retinal sensitivities in all areas with either MP-3 or HFA were significantly and strongly correlated with EZ length (Fig S5A, available at www.ophthalmologyscience.org).

### Longitudinal Correlation between Anatomical and Functional Metrics in Patients with RP

We next analyzed the longitudinal relationships between MP-3 and HFA values and EZ lengths. The results are summarized in Table 3. The slopes of mean retinal sensitivities with MP-3 at the radii of 2°,4°, and 8° were −0.60 dB/year, −0.67 dB/year, and −0.62 dB/year, respectively. The slopes of mean retinal sensitivities with HFA at the radii of 2° and 4° and 8° were −0.40 dB/year, −0.54 dB/year, and 0.60 dB/year, respectively. The slope of EZ length was −88.92 μm/year.

**Table 3:** The Annual Changes of Anatomical and Functional Metrics in Patients with Retinitis Pigmentosa

Each Metric	Mean Value ± Standard Error	*P* Value
MP-3		
Mean retinal sensitivity		
Radius of 2°, dB/yr	−0.60 ± 0.12	<0.0001
Radius of 4°, dB/yr	−0.67 ± 0.10	<0.0001
Radius of 8°, dB/yr	−0.62 ± 0.07	<0.0001
HFA		
Mean retinal sensitivity		
Radius of 2°, dB/yr	−0.40 ± 0.12	0.002
Radius of 4°, dB/yr	−0.54 ± 0.10	<0.0001
Radius of 8°, dB/yr	−0.60 ± 0.11	<0.0001
OCT		
EZ length, μm/yr	−88.92 ± 12.71	<0.0001

dB = decibels; EZ = ellipsoid zone; HFA = Humphrey Field Analyzer; MP-3 = microperimetry-3.

**Figure 6:**
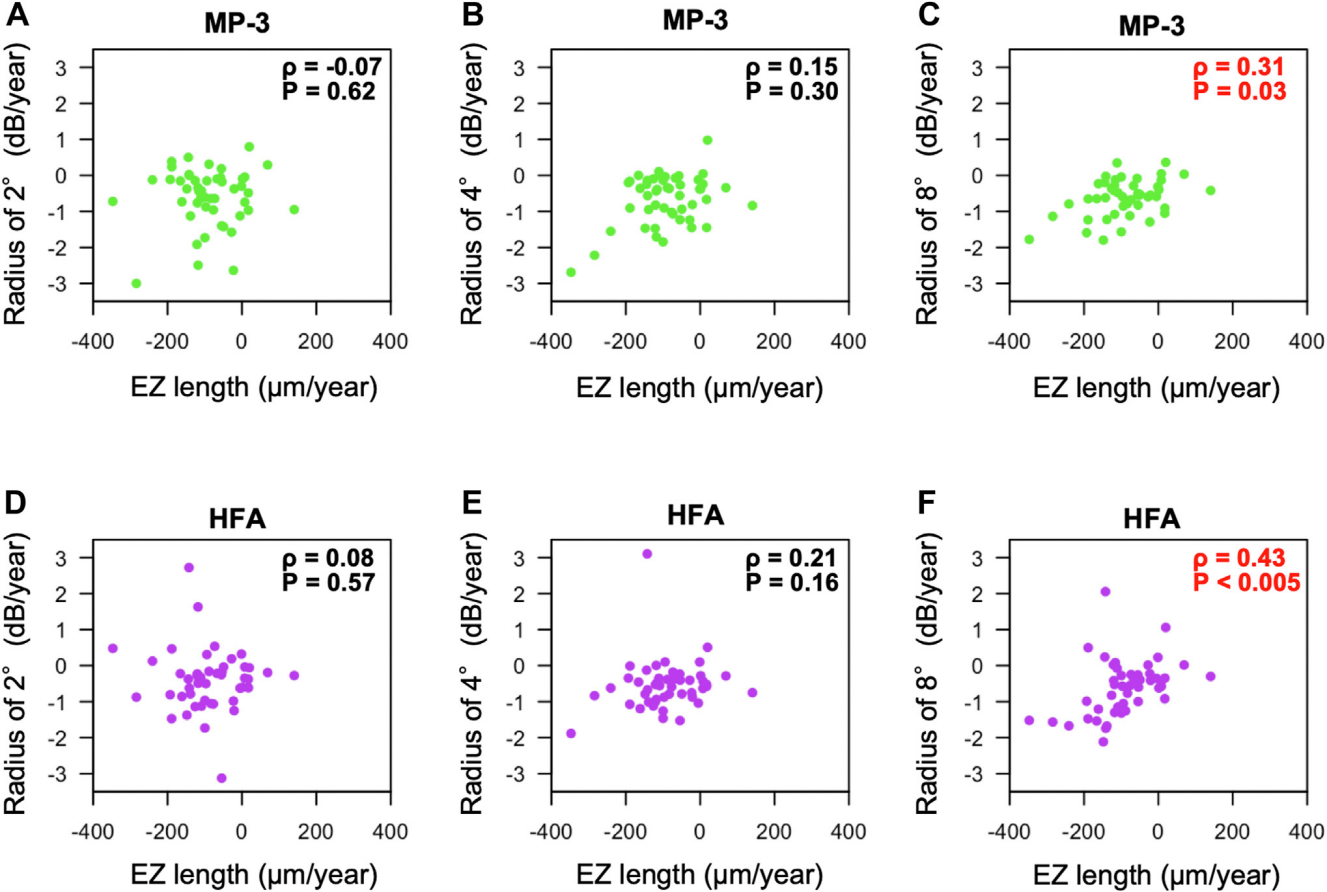
Relationships between the regression coefficients of EZ length and those of retinal functional parameters in the longitudinal RP patient data. Scatter plots show the associations of EZ length slope with the mean retinal sensitivity slope of MP-3 at the radius 2° (**A**), 4° (**B**), and 8° (**C**), and the mean retinal sensitivity of HFA at the radius 2° (**D**), 4° (**E**), and 8° (**F**). Green dots indicate MP-3 data, and purple dots indicate HFA data. dB = decibels; EZ = ellipsoid zone; HFA = Humphrey Field Analyzer; MP-3 = microperimetry-3; RP = retinitis pigmentosa.

**Figure 7:**
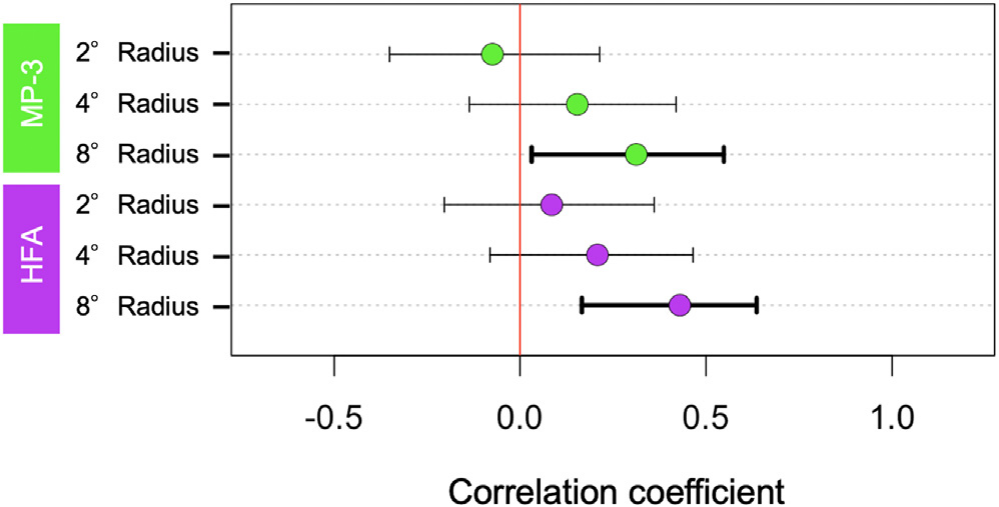
Correlation coefficients at the radii of 2°, 4°, and 8° in longitudinal data. Green and purple dots show correlation coefficients between the slopes of EZ length and retinal sensitivities measured by MP-3 or HFA at the radii of 2°, 4°, and 8°. Bars show 95% confidence interval bounds for each correlation coefficient. The slopes of retinal sensitivities at the radius of 8° with either MP-3 or HFA were significantly correlated with those of EZ length. The correlation coefficients were analyzed by Spearman’s rank correlation test. EZ = ellipsoid zone; HFA = Humphrey Field Analyzer; MP-3 = microperimetry-3.

The correlation coefficients between the slope of EZ length and the slope of retinal sensitivities at the radii of 2°, 4°, and 8° measured with MP-3 were −0.07 (*P* = 0.62), 0.15 (*P* = 0.30), and 0.31 (*P*=0.03), and those between the slope of EZ length and the slope of retinal sensitivities at the radii of 2°, 4°, and 8° measured with HFA were 0.08 (*P* = 0.57), 0.21 (*P* = 0.16), and 0.43 (*P* < 0.005), respectively (Fig 6). The correlation coefficients with their 95% confidence intervals are shown in Figure 7. The EZ length slope was significantly correlated with the retinal sensitivity slope at the radius of 8° measured with MP-3 and HFA, but it was not correlated with the retinal sensitivity slope at the radius of 2° or 4°.

With the segmentation of foveal, parafoveal, and perimacular areas, retinal sensitivity slope in the perimacular area, measured by both MP-3 and HFA, were significantly correlated with EZ length slope. The retinal sensitivity slope in the foveal and parafoveal area, measured by HFA but not MP-3, was also significantly correlated with EZ length slope (Fig S5B, available online at www.ophthalmologyscience.org).

### Effects of Fixation Stability on the Structure–Function Correlation with MP-3 or HFA in Patients with RP

**Figure 8:**
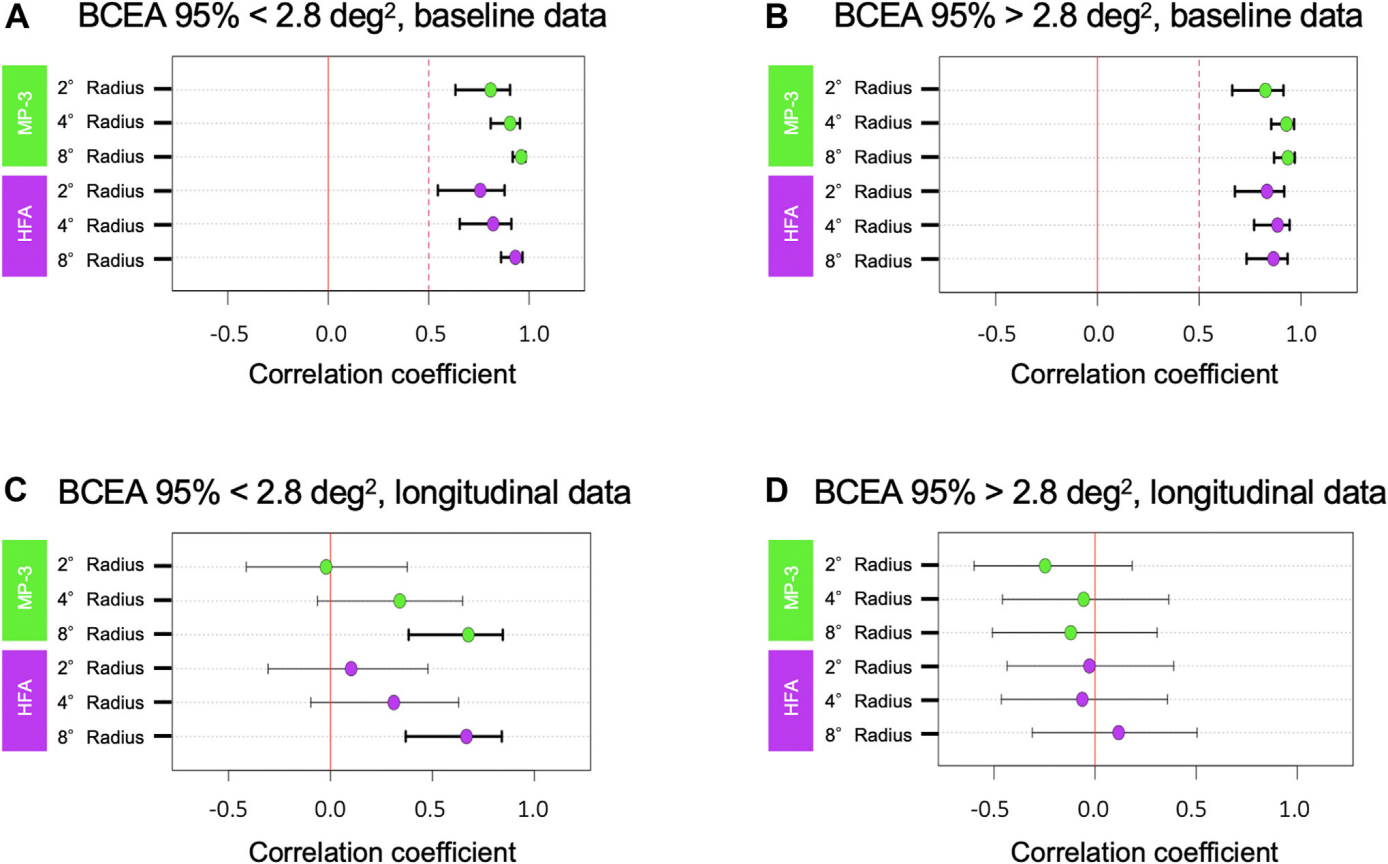
Subgroup analysis of correlation coefficients by fixation stability. Green and purple dots show correlation coefficients between EZ length and retinal sensitivities measured by MP-3 or HFA at the radii of 2°, 4°, and 8° in cross-sectional baseline data (**A** and **B**) or in longitudinal data (**C** and **D**) in the better fixation stability (BCEA 95% < 2.8 deg^2^) group (**A** and **C**) or in worse fixation stability (BCEA 95% >2.8 deg^2^) group (**B** and **D**). Bars show 95% confidence interval bounds for each correlation coefficient. The correlation coefficients were analyzed by Spearman’s rank correlation test. BCEA = bivariate contour ellipse area; EZ = ellipsoid zone; HFA = Humphrey Field Analyzer; MP-3 = microperimetry-3.

Microperimetry-3 provides a measure of fixation such as bivariate contour ellipse area (BCEA), and previous studies on BCEA 95% using MAIA in healthy subjects and patients with macular diseases including patients with RP showed that BCEA 95% <4 deg^2^ was an ideal cutoff value indicating high test reliability.^38^ In this study, mean BCEA 95% at baseline was 2.8 ± 1.7 deg^2^, suggesting that participants had good fixation stability. To analyze our data in terms of fixation stability, we divided the patients into lower (better) and higher (worse) BCEA 95% groups, with the mean value of 2.8 deg^2^. In the cross-sectional analysis, strong structure–function correlation was observed in both BCEA groups (Fig 8A, B). However, in the longitudinal analysis, retinal sensitivity slope only in the lower BCEA group was significantly correlated with EZ length slope, for both MP-3 and HFA (Fig 8C, D).

## Discussion

In the present study, we evaluated macular function and progression of central vision loss in patients with RP using OCT, MP, and SAP. Cross-sectional analysis revealed a high and significant correlation between EZ length and retinal sensitivities measured using either MP-3 or HFA instruments at all the areas examined (i.e., 2°, 4°, and 8°). In contrast, in longitudinal analysis, the function–structure correlation was weaker, and only the retinal sensitivity slope at the radii of 8° with MP-3 and HFA was significantly correlated with the EZ length slope. The performances of HFA and MP-3 were comparable in evaluating macular status and disease progression in RP.

In cross-sectional analysis, previous studies suggest that MP exhibits better structure–function correlation than SAP in patients with RP. The MAIA showed a correlation coefficient of 0.68 between retinal sensitivity at the radius of 16° and EZ area,^8^ while Octopus showed a correlation coefficient of 0.48 between total hill of vision (volumetric measure for retinal sensitivity at the radius of 80°) and central foveal thickness in patients with usherin-related RP.^15^ Moreover, Asahina et al demonstrated that the retinal sensitivities measured by MP-3 were significantly correlated with the EZ intact area, while those measured by HFA showed no association with the EZ intact area.^7^ However, our study, which had a larger sample size, showed significant structure–function correlation in the cross-sectional analysis by both MP-3 and HFA tests, with the degree of correlation being comparable between the 2 modalities. Our data also showed that retinal sensitivities in the radii of 4° and 8°, rather than 2°, were better associated with EZ length.

Compared with the cross-sectional analysis, the longitudinal analysis in our study exhibited weaker structural-function correlation. There was 1 study which analyzed the longitudinal association between structure and function in patients with RP, and the correlation coefficient between EZ area and Octopus mean sensitivity was 0.38 (95% confidence interval: 0.18, 0.55) in 2-year analysis of patients with usherin-related RP.^16^ This correlation coefficient was consistent with that using MP-3 (ρ = 0.31, *P* = 0.03) or HFA (ρ = 0.43, *P* < 0.005). There are 2 explanations for this observation. First, the frequency (3.3 times on average) and period (35.5 months on average) of examinations may not have been sufficient to precisely evaluate the progression of RP patients. Our study showed that the standard error from the regression line was 0.12, 0.10, and 0.11 dB/year for HFA at the 2°, 4°, and 8° radii, respectively; in contrast, the mean rate of retinal sensitivity loss in the HFA 10-2 tests was 0.40, 0.54, and 0.60 dB/year at the 2°, 4°, and 8° radii, respectively. Similarly, the standard error from the regression line was 0.12, 0.10, and 0.07 dB/year for MP-3 at the 2°, 4°, and 8° radii, respectively; in contrast, the mean rate of retinal sensitivity loss in the MP-3 test was 0.60, 0.67, and 0.62 dB/year at the 2°, 4°, and 8° radii, respectively. Therefore, to draw a reliable regression line of retinal sensitivity loss in patients with RP, there may be a need for more frequent examinations, such as were conducted in the United Kingdom Glaucoma Treatment Study trial evaluating the effect of latanoprost on visual field preservation in patients with glaucoma (16 HFA tests were scheduled over 24 months).^33^ Indeed, in a phase III study evaluating the efficacy of oral N-acetylcysteine for RP (NAC Attack study), the investigators have scheduled 7 examinations over 45 months.

Second, there is a possibility that the progression phase is different between structural and anatomical measures. It was reported that disease progression in functional tests is faster in the middle to late stage of RP,^17^^,^^18^ while that in anatomical tests can be better detected in the early stage.^27^ Our present study included patients in various stages of RP, from early to late stages, which may have resulted in the lower structure–function relationships for RP progression.

In longitudinal analysis, our data also showed that RP patients with better BCEA 95% (<2.8 deg^2^) exhibited higher structure–function correlation for both HFA and MP-3, suggesting that patients with RP with good fixation stability are suitable for evaluating disease progression with either MP or SAP. Previous studies reported better test–retest reliability and structure–function correlation with MP-3, equipped with real-time tracking, in patients with RP.^6^^,^^7^ Contrary to our expectation, MP did not show a significant advantage over SAP, even when analyzed in the subgroup with worse fixation stability. The benefit from real-time fundus tracking may be more pronounced in patients with worse fixation stability.

There were several limitations in this study. First, the more frequent examinations and longer follow-up period may have led to better evaluation of RP progression, as mentioned above. Second, this was a single-center study with a relatively small number of participants. Thirdly, it has been shown MP sensitivity values are affected by age and loci^39^ and this needs to be taken into account in data interpretation. Finally, the causative genes were identified in only 39.6% of study participants, and therefore the correlation between RP progression and genetic variants was not analyzed in this study. Despite these limitations, this study directly compares the utility of MP-3 and HFA for assessing RP progression, and the results may provide important information for future clinical studies.

In conclusion, functional parameters measured with MP-3 and HFA showed comparable correlation with the anatomical status in patients with RP; however, the correlations became weaker in longitudinal analysis than in cross-section analysis. For evaluating the progression of RP, the retinal sensitivity loss at 8° radius was best associated with EZ length progression. These data suggest that researchers need to explore more sensitive outcome measures to evaluate RP progression for future clinical trials that aim to preserve macular function.
