# Supplementary material for: Comparison of Microperimetry and Static Perimetry for Evaluating Macular Function and Progression in Retinitis Pigmentosa
Source: Ophthalmol Sci. 2024 Jul 20;4(6):100582. doi: 10.1016/j.xops.2024.100582 (PMC11388686; doi:10.1016/j.xops.2024.100582)
Supplement: Figure S4 [file mmc1.pdf]

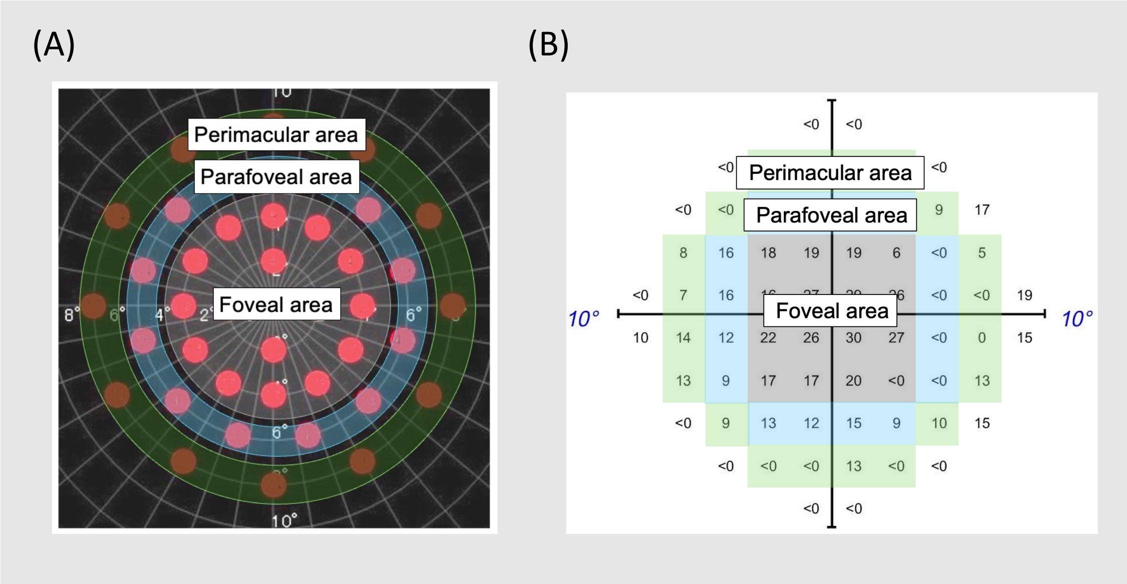

Figure S4 . Methods to evaluate the macular function with MP-3 and HFA in similar number of loci between the 3 rings

(A) In MP-3 data analysis, retinal sensitivity loci was divided into foveal area (central 16 points), parafoveal area (central 12 points), and perimacular area (central 12 points).

(B) In HFA data analysis, retinal sensitivity loci was divided into foveal area (central 16 points), parafoveal area (central 16 points), and perimacular area (central 20 points).

MP-3: Microperimetry-3; HFA: Humphrey field analyzer; OCT: optical coherence tomography; EZ: ellipsoid zone.
