# Supplementary material for: Comparison of Microperimetry and Static Perimetry for Evaluating Macular Function and Progression in Retinitis Pigmentosa
Source: Ophthalmol Sci. 2024 Jul 20;4(6):100582. doi: 10.1016/j.xops.2024.100582 (PMC11388686; doi:10.1016/j.xops.2024.100582)
Supplement: Figure S5 [file mmc2.pdf]

(A)

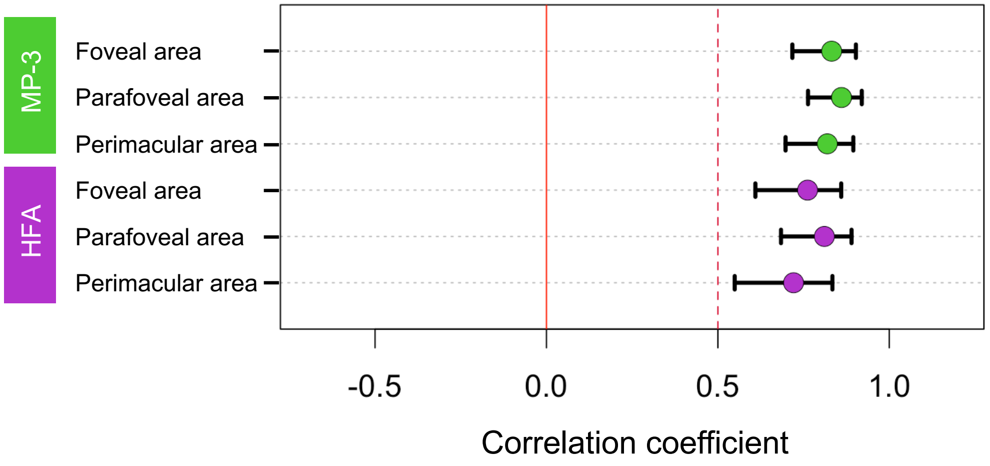


(B)

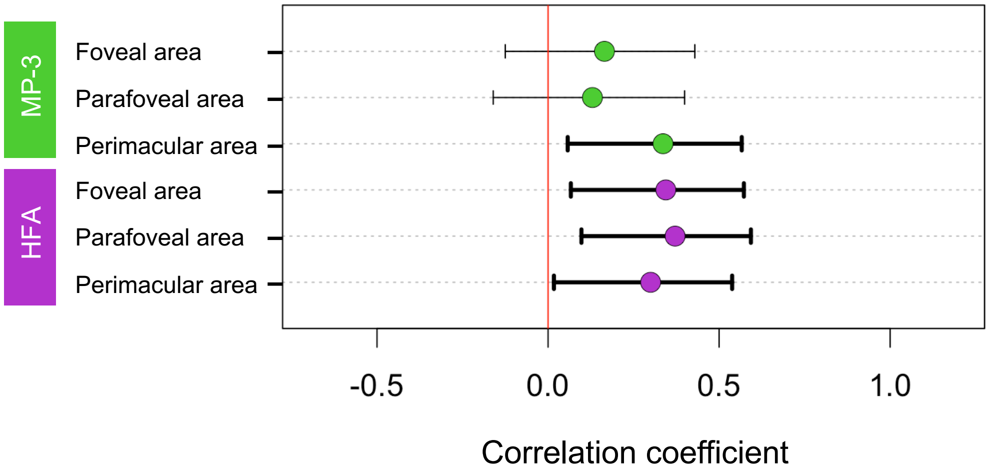


**Figure S5. Correlation coefficients within foveal, parafoveal, and perimacular area in the cross-sectional and longitudinal data**

Green and purple dots show correlation coefficients between EZ length and retinal sensitivities measured by MP-3 or HFA in foveal, parafoveal, and perimacular area in cross-sectional baseline data (A) and in longitudinal data (B). Bars shows 95% confidence interval bounds for each correlation coefficient. The correlation coefficients were analyzed by Spearman's rank correlation test.

MP-3: Microperimetry-3; HFA: Humphrey field analyzer; EZ: ellipsoid zone
